# Continuous vs. interrupted suturing in hepaticojejunostomy: a comprehensive systematic review and meta-analysis

**DOI:** 10.1007/s00423-025-03756-y

**Published:** 2025-07-04

**Authors:** Ahmed Abdelsamad, Mohammed Khaled Mohammed, Ibrahim Khalil, Zeyad M. Wesh, Omar A. Ahmed, Ahmed Elsherif, Jawad J. F. Alqedra, Khaled Ashraf Mohamed, Eslam Elmaghraby, Torsten Herzog, Florian Gebauer

**Affiliations:** 1 Surgery Department II, University of Witten/Herdecke, 58455 Witten, Germany; 2 Deputy Head of Oncological Surgery- Section Head of Robotic Surgery, Knappschaft Vest Hospital, 45657 Recklinghausen, Germany; 3 Faculty of Medicine, Cairo University, Cairo, Egypt; 4 Faculty of Medicine, Alexandria University, Alexandria, Egypt; 5 Faculty of Medicine, Ain Shams University, Cairo, Egypt; 6 St. Josef Hospital Bochum, Bochum, Germany; 7 General Surgery Department, Cairo University, Giza, Egypt; 8 Department of Surgery (A), Heinrich-Heine University Hospital Düsseldorf -UKD, 40225, Düsseldorf, Germany; 9 Surgery Department, Ruhr University Bochum, Bochum, Germany; 10 Head of Surgery Department, Helios University Hospital, Wuppertal, Germany

**Keywords:** Continuous sutures, Interrupted sutures, Hepaticojejunostomy, Surgical outcomes, Meta-analysis

## Abstract

**Background:**

Hepaticojejunostomy (HJ) is a crucial reconstructive step in upper gastrointestinal (UGI), pancreaticoduodenectomy (PD), and Hepaticobiliarypancreatic (HBP) surgeries. The optimal suturing technique remains debated, with conflicting evidence regarding operative efficiency, costs, and complications. This meta-analysis compares continuous and interrupted suturing to provide evidence-based recommendations.

**Methods:**

A systematic review and meta-analysis were conducted using PubMed, Embase, and Cochrane Library. Primary outcomes were anastomotic time and costs, while secondary outcomes included bile leakage, anastomotic stricture, morbidity, cholangitis, hospital stay, and re-exploration rates. A random or fixed-effects model was applied based on heterogeneity. We included randomized controlled trials and non-randomized cohort studies. The risk of bias was assessed using the Cochrane ROB 2 tool, Newcastle–Ottawa Scale (NOS), and MINORS instrument as appropriate. Additionally, the quality of evidence for each outcome was evaluated using the GRADE approach. Sensitivity analyses were performed using the leave-one-out method.

**Results:**

Seven studies (1,159 patients) were included (continuous: 388, interrupted: 771). Continuous suturing significantly reduced anastomotic time (MD = -13.06 min, 95% CI: -17.37 to -8.75, *P* < 0.001) and costs (SMD = -4.89, 95% CI: -6.10 to -3.67, *P* < 0.001). However, no significant differences were observed in bile leakage, anastomotic stricture, morbidity, cholangitis, hospital stay, or re-exploration rates (*P* > 0.05). Sensitivity analyses confirmed these findings.

**Conclusion:**

Continuous suturing reduces anastomotic time by ~ 13 min and costs by ~ $90 without increasing complications. While these differences may be statistically significant, their clinical relevance can vary depending on the surgical context. Surgical choice should consider surgeon expertise, institutional protocols, and patient factors. Further randomized controlled trials are necessary to validate these findings.

**Supplementary Information:**

The online version contains supplementary material available at 10.1007/s00423-025-03756-y.

## Introduction

Hepaticojejunostomy (HJ) is a critical reconstructive procedure in pancreaticoduodenectomy (PD), upper gastrointestinal tract (GIT) surgeries, and HBP surgeries to restore bile flow after resection [1]. The technique used for anastomosis plays a crucial role in determining surgical efficiency, anastomotic integrity, and postoperative outcomes [2].

Surgeons traditionally debate between continuous and interrupted suturing, with both approaches having theoretical advantages and limitations [3, 4]. Continuous suturing is often advocated for its technical simplicity and reduced operative time especially in the wide common hepatic duct **(CHD)** [5], while interrupted suturing is considered to provide more secure anastomotic healing, potentially reducing complications [6–8].

However, the superiority of one technique over the other remains controversial.

Several studies have examined the impact of suturing techniques on hepaticojejunostomy outcomes, with conflicting results [5, 9–15].

Some reports suggest that continuous suturing reduces operative time and overall surgical burden without increasing complications [9, 10, 12], whereas others emphasize concerns about anastomotic integrity and the potential for strictures or leaks [11, 13–15]. Saxane et al. (2021) [11] have proposed a size-based approach for selecting the suturing technique, using 8 mm as the threshold for switching between interrupted and continuous suturing.

Additionally, Seifert et al. [13]reported significantly longer suture times in the interrupted suturing group compared to the continuous group (median 22.4 vs. 12.0 min; *P* < 0.001), while short- and long-term biliary complication rates, including bile leakage and anastomotic stenosis, were comparable between groups.

 Similarly, Tatsuguchi et al. [9] demonstrated that interrupted suturing significantly increased both anastomosis time and cost compared to continuous suturing (27.0 ± 6.6 min vs. 16.2 ± 5.0 min; $144.7 ± 34.6 vs. $11.7; *p* < 0.001) [9].

Moreover, the economic implications of each technique remain unclear, with some evidence pointing to cost reductions associated with continuous suturing [9, 10, 12, 13]. At the same time, other studies argue that any initial cost savings may be offset by postoperative complications requiring further intervention [11, 14–17].

Given the lack of consensus in the literature, there is a need for a comprehensive synthesis of the available evidence to determine the optimal suturing technique for hepaticojejunostomy in pancreaticoduodenectomy. The aim of this systematic review and meta-analysis is to evaluate and compare continuous and interrupted suturing techniques in terms of their impact on operative efficiency, postoperative complications, and clinical outcomes. By consolidating existing data, we seek to provide evidence-based recommendations that may guide surgical decision-making and improve patient outcomes in hepatobiliary and pancreatic surgery.

## Methods

### Data sources and searches

We performed a systematic search of online databases, including PubMed, Scopus, Embase, Cochrane Library, and Web of Science, from inception up to the first of February, 2025. The search terms were designed to capture key concepts related to our study. Our study idea commenced on the third of January 2025.

### Study design and outcomes (PICOS Framework)

This systematic review and meta-analysis was developed based on the PICOS framework, which defines the study elements as follows:

- Population (P): Adult patients (≥ 18 years) undergoing hepaticojejunostomy after pancreaticoduodenectomy for benign or malignant disease.
- Intervention (I): Continuous suturing technique.
- Comparison (C): Interrupted suturing technique.
- Outcomes (O): The primary outcomes were anastomotic time and costs. The secondary outcomes included bile leakage, anastomotic stricture, morbidity (short-term and overall), cholangitis, hospital stay, re-exploration rates, and overall survival.
- Study Design (S): Randomized controlled trials, cohort studies, and cross-sectional studies providing comparative data.
- This framework guided our study selection, data extraction, and synthesis of findings.

Cholangitis was extracted as a reported complication; however, no included study defined it explicitly or referenced standardized diagnostic criteria. For context, the Tokyo Guidelines [18] define acute cholangitis based on clinical evidence of systemic inflammation (e.g., fever, leukocytosis), cholestasis (elevated bilirubin or liver enzymes), and imaging findings indicative of biliary obstruction. In the absence of standardization across studies, we extracted cholangitis data as reported by the original authors.

### Eligibility criteria

We included full-text English articles published in international peer-reviewed journals comparing continuous versus interrupted suturing techniques for hepaticojejunostomy (HJ) following UGI and Hepaobiliarypancreatic (HBP) surgery. Studies had to provide sufficient data for both qualitative and quantitative analysis, including at least two relevant outcomes for this systematic review and meta-analysis.

Studies were included if they reported on patients undergoing HJ following HBP surgery for benign or malignant diseases, provided comparative data between continuous and interrupted suturing techniques like safety, cost, and Anastomotic time, and reported perioperative or long-term clinical outcomes. Only studies involving adult patients (aged 18 years or older) were considered.

Exclusion criteria included studies that did not compare both suture techniques as single-arm studies, studies including patients who underwent open surgical procedures without reporting on HJ, studies presenting mixed or combined suturing approaches, studies on experimental models (animal or cadaveric), narrative or systematic reviews, meta-analyses, video reports, case reports, case series with fewer than 10 patients per group, and abstract-only publications. Duplicate studies and those without extractable data were also excluded.

### Search strategy

**Fig. 1:**
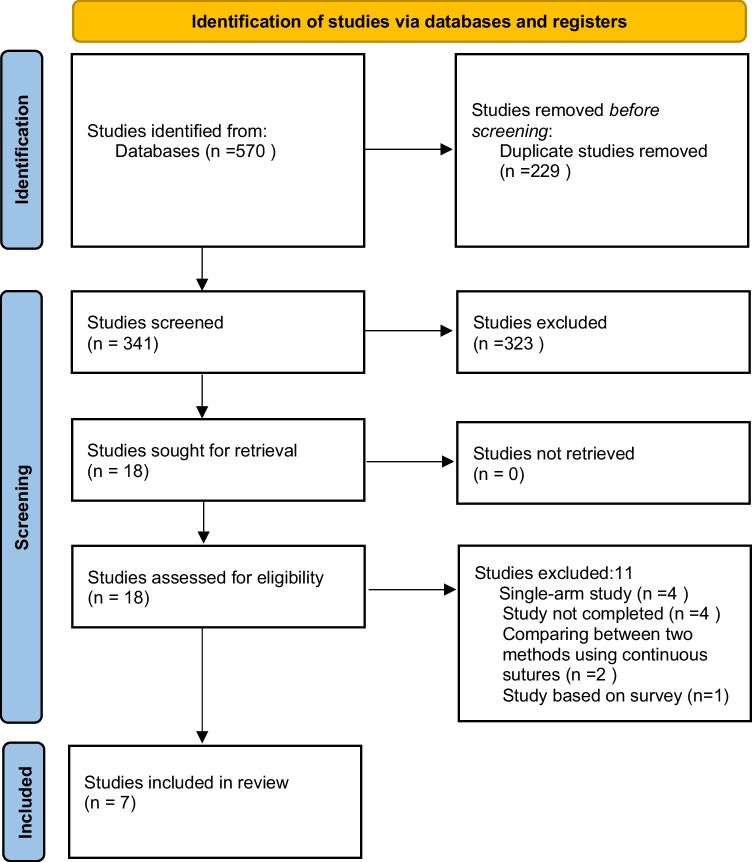
PRISMA 2020 flow diagram for new systematic reviews which included searches of databases and registers only. Source: Page MJ, et al. BMJ 2021;372:n71. 10.1136/bmj.n71. This work is licensed under CC BY 4.0. To view a copy of this license, visit https://creativecommons.org/licenses/by/4.0/

**Fig. 2:**
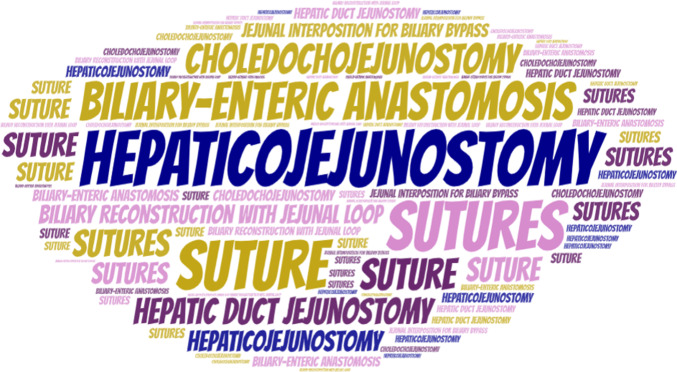
Word cloud of the MeSH-NCBI search keywords

This systematic review and meta-analysis followed the PRISMA guidelines and Cochrane Collaboration recommendations, as presented in Fig. 1. A comprehensive literature search was conducted using Medline (via PubMed), Web of Science, and Cochrane database using “title and abstract” as described in the Cochrane Handbook for Systematic Reviews of Interventions (chapter 4.4.4) [19], up to February 1, 2025. The search strategy included controlled terms and synonyms from the MeSH database such as ‘Hepaticojejunostomy,’ ‘Biliary-enteric anastomosis,’ ‘suture,’ and ‘sutures’ to ensure broad retrieval of relevant studies. A detailed search strategy is provided in Table 1. The Word cloud of the MeSH-NCBI search keywords is shown in Fig. 2.

**Table 1:** MeSH and Emtree terms (Database search)

Database	Search term	Filter	Results
Embase	(sutures OR suture) AND (hepaticojejunostomy OR"Biliary-enteric anastomosis"OR choledochojejunostomy OR"Hepatic duct jejunostomy"OR"Biliary reconstruction with jejunal loop"OR"Jejunal interposition for biliary bypass")	Title/Abstract	23
SCOPUS	TITLE-ABS (sutures OR suture) AND TITLE-ABS (hepaticojejunostomy OR"Biliary-enteric anastomosis"OR choledochojejunostomy OR"Hepatic duct jejunostomy"OR"Biliary reconstruction with jejunal loop"OR"Jejunal interposition for biliary bypass")	Title/Abstract	171
WOS	TS = ((sutures OR suture) AND (hepaticojejunostomy OR"Biliary-enteric anastomosis"OR choledochojejunostomy OR"Hepatic duct jejunostomy"OR"Biliary reconstruction with jejunal loop"OR"jejunal interposition for biliary bypass"))	Topic	203
PubMed	((suture[Title/Abstract]) OR (sutures[Title/Abstract])) AND ((((Hepaticojejunostomy[Title/Abstract]) OR ("Biliary-enteric anastomosis"[Title/Abstract])) OR (Choledochojejunostomy[Title/Abstract])) OR ("Hepatic duct jejunostomy"[Title/Abstract]))	Title/Abstract	173

Four independent reviewers (J.J.F, A.E, O.A.A, and Z.W) screened the retrieved articles by title and abstract, followed by full-text evaluation. Disagreements were resolved through discussion or a fifth reviewer's (MKM) decision.

### Data extraction

Data were extracted into a standardized Microsoft Excel spreadsheet. Extracted data included study characteristics (authors, publication year, study design, sample size), and patient demographics, as per Table 2. Perioperative variables (Anastamosis time, blood loss, postoperative complications, bile leak rates), and long-term outcomes (stricture rates, reoperation rates) were also gathered. Operative morbidity was assessed according to the Clavien–Dindo classification system, where available, to ensure standardized reporting and comparability across studies [20].

**Table 2:** Characteristics of included studies. This table summarizes key information on study origin, patient demographics, diagnoses, and operative procedures for each included study evaluating continuous versus interrupted suturing in hepaticojejunostomy

Author (Year)	Country	Patients(n)	Age (years)	Sex (M/F)	BMI (kg/m^2^)	Diagnosis	Operative Procedure
Brunner et al. [5]	Germany	100 (50 patients in each group)	67(12)	65 (35)	26.3 (5..6)	Pancreatic adenocarcinoma (49%)Other malignant pancreatic head tumors (29%)Chronic pancreatitis (12%)Cystic pancreatic neoplasms (10%)	Elective open partial pancreaticoduodenectomy with hepaticojejunostomy72% underwent pylorus-preserving pancreaticoduodenectomy (PPPD)28% underwent classical Whipple procedure15% required venous vascular resection
Tatsuguishi et al. [9]	Japan	161	65.2 ± 9.4	(65.2 ± 9.4)	NA	Benign or malignant pathologies of the pancreas, extrahepatic bile duct, or periampullary region.	Total pancreatectomy and Pancreaticoduodenectomy
Saxane et al. [11]	India	556	47.45 ± 15.205	(228/328)	21.55 ±3.055	All procedures requiring biliary-enteric anastomosis	Hepaticojejunostomy
Natsume et al. [12]	Japan	172	64(29.5-81.5)	(105/67)	22.45(16.4-34.85)	Periampullary diseases	Pancreatoduodenectomy and Hepaticojejunostomy
Seifert et al. [13]	Germany	80	69.5(64.25-76.5)	(37/43)	24.6(25-26)	All procedures requiring biliary-enteric anastomosis	Hepaticojejunostomy or Choledochojejunostomy
Sabra et al. [14]	Egypt	57	6.01	(20/37)	24.18	Choledochal cyst	Hepaticojejunostomy/ Hepaticoduodenostomy
Yadav et al. [15]	Nepal	34	48 IS/ 53.2 CS	(26/08)	20.7 IS/ 19.5 CS	Benign 9 IS/ 3 CS // Malignant 9IS/ 13 CS	Hapaticojejunostomy

For continuous outcomes, the choice between reporting the Mean Difference (MD) or the Standardized Mean Difference (SMD) depended on the measurement scales used across studies. The MD was used when all studies included in a specific analysis measured the outcome using identical scales and units. The SMD (calculated as Hedges'g to correct for small sample bias) was used when studies assessed the same construct using different scales, allowing for pooling of the results [21].

## Quality assessment

### Risk of bias assessment

**Fig. 3:**
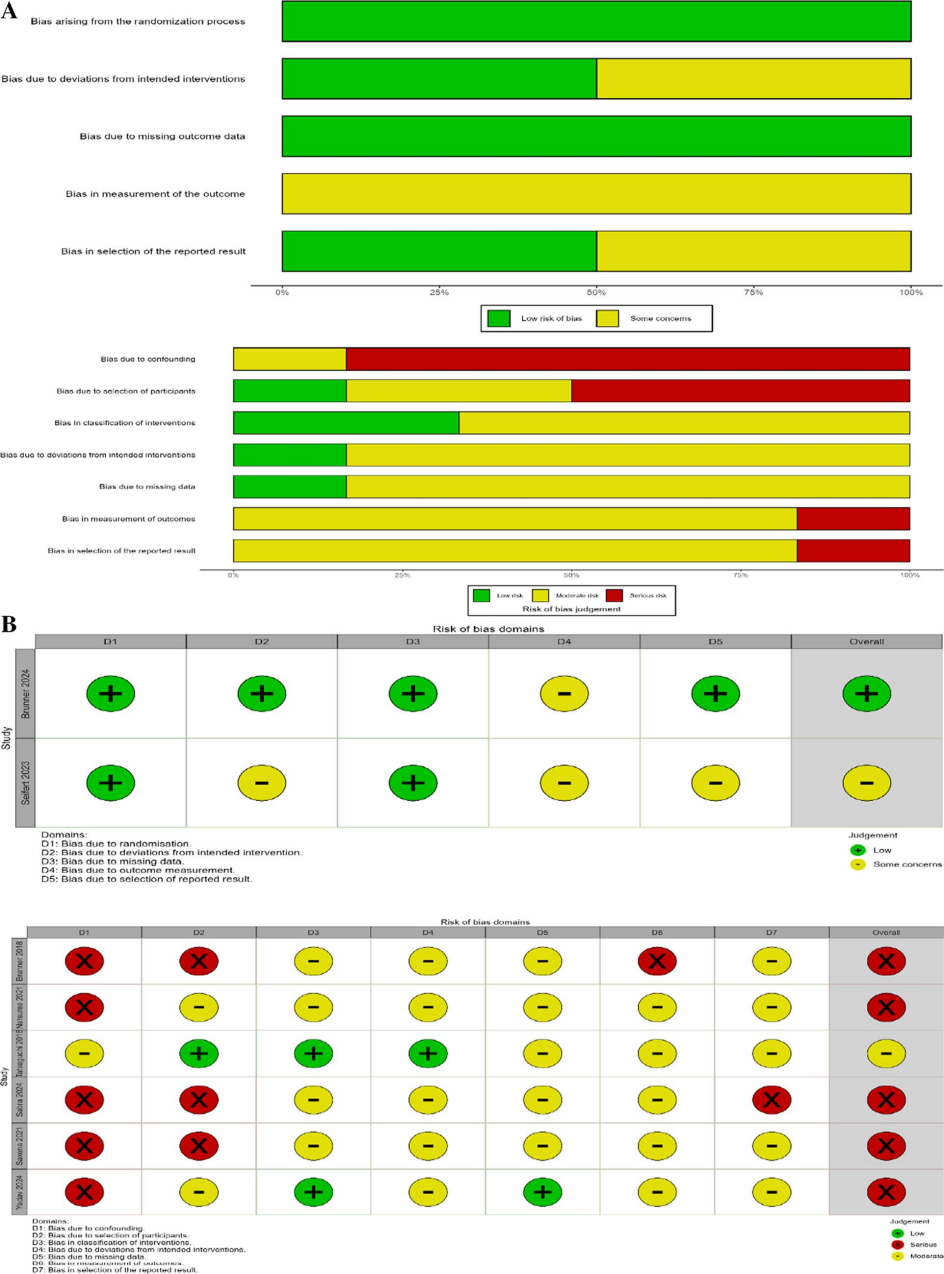
Risk of bias assessment across included studies**.** Risk of bias was assessed for randomized controlled trials using the Cochrane Risk of Bias Tool (version 1.0), and for non-randomized studies using the ROBINS-I tool. The stacked bar charts display the proportion of studies with low, moderate, or high/serious risk of bias in each domain. Summary tables below indicate the judgment for each study across key domains. Green circles denote low risk of bias, yellow circles indicate some concerns or moderate risk, and red circles represent serious or high risk of bias

We conducted a comprehensive risk of bias assessment using standardized tools. For randomized controlled trials, we applied the Cochrane Risk of Bias tool 2.0 (RoB 2) [22], which evaluates five domains: randomization process, deviations from intended interventions, missing outcome data, measurement of outcomes, and selective reporting. For non-randomized studies, we employed the Risk of Bias in Non-randomized Studies of Interventions (ROBINS-I) tool, which assesses seven domains: confounding, selection of participants, classification of interventions, deviations from intended interventions, missing data, measurement of outcomes, and selection of reported results [23], as per (Fig. 3).

Two independent reviewers performed the assessment, with discrepancies resolved through discussion. The evaluation was visualized using the'robvis'package in R software (version 4.1.0) to generate traffic light plots and summary graphs.

## Assessment of quality of evidence

The GRADE (Grading of Recommendations Assessment, Development, and Evaluation) system was used. It was applied to each outcome included in our meta-analysis. Considering factors such as study design, result consistency, estimation precision, potential biases, and clinical relevance of the findings [24].

### Statistical analysis

The analysis utilized R Studio (version 2024.09.0, Build). For continuous outcomes (anastomotic time, costs, and length of hospital stay), we calculated mean differences (MD) or standardized mean differences (SMD) with 95% confidence intervals (CIs) using random-effects models when substantial heterogeneity was present (I^2^ ≥ 90%) and fixed-effects models when heterogeneity was low [25, 26]. For dichotomous outcomes (anastomotic stricture, bile leakage, morbidity, survival, cholangitis, and re-exploration rates), we calculated risk ratios (RR) with 95% CIs using Mantel–Haenszel fixed-effect models [27], applying a 0.5 continuity correction only for studies with zero events [28]. Heterogeneity was assessed using I^2^ statistics, which indicates the proportion of variability due to heterogeneity and was not solely used to guide model selection. The model choice was predefined based on outcome type and expected variability. A random-effects model (DerSimonian and Laird) was used for continuous outcomes due to anticipated heterogeneity. For dichotomous outcomes, a fixed-effect model was applied when heterogeneity was absent (I^2^ = 0%, non-significant Q test). Heterogeneity measures (I^2^, Tau^2^, Q test) supported the interpretation[29]. We also conducted sensitivity analyses using the leave-one-out method to evaluate the robustness of our findings and identify potential sources of heterogeneity [30]. Statistical significance was set at *P* < 0.05. All analyses were performed using R (version unspecified) with the meta [31], metaphor [26], and forestplot [32] packages. Forest plots were generated to visualize effect sizes and their precision, with studies weighted according to inverse variance for continuous outcomes and Mantel–Haenszel weights for dichotomous outcomes.

## Results

Following the PRISMA flowchart and our predefined inclusion criteria, **seven studies** [5, 9, 11–15] fulfilled the eligibility requirements and were included in the final meta-analysis. These studies encompassed both randomized controlled trials and non-randomized cohort designs, providing comparative data on continuous versus interrupted suturing in hepaticojejunostomy following HBP surgery. **A total of 1,159** patients were analyzed across all included studies (Table 3).

**Table 3:**
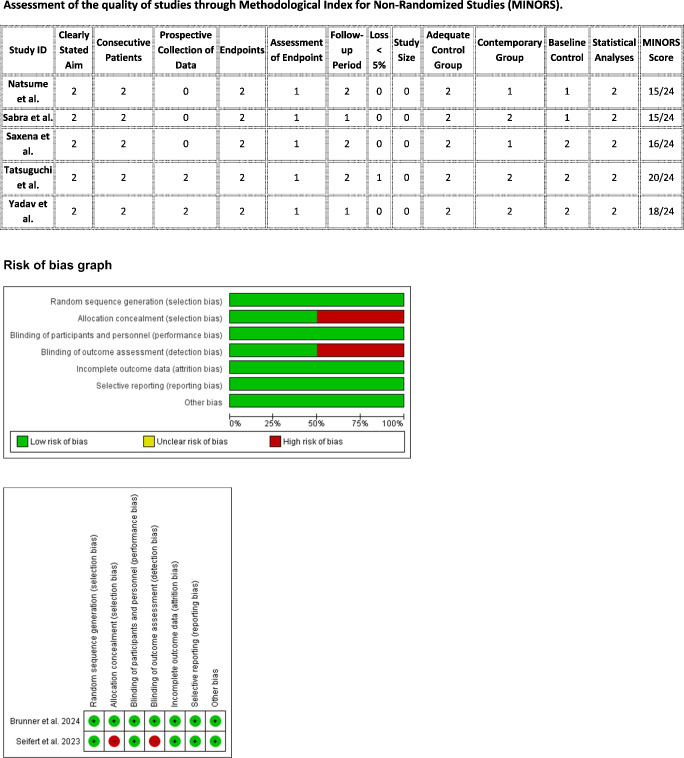
Risk of bias and methodological assessment summary

As detailed in Table 2, the included studies encompass a range of patient populations undergoing biliary-enteric anastomosis in the context of both benign and malignant conditions affecting the pancreas, extrahepatic bile ducts, and periampullary region. Operative procedures included hepaticojejunostomy, choledochojejunostomy, hepaticoduodenostomy, and various forms of HBP surgeries.

All procedures across the included studies were performed using an open surgical approach. No study reported the use of minimally invasive surgery (MIS), or robotic surgery.

 Notably, the study by Saxane et al. (2021) [ 11 ] reported significant differences in intraoperative parameters between the interrupted and continuous suturing groups. Vicryl was the predominant suture material used, and the interrupted group included significantly more patients than the continuous group (363 vs. 24 patients; *P* < 0.001), and a higher number of sutures were applied (13 vs. 2; *P* < 0.001). This was associated with a significantly longer anastomotic time (45 min vs. 21 min; *P* < 0.001) and higher suturing cost ($104 vs. $17; *P* < 0.001). These unique procedural characteristics may partly explain the heterogeneity observed in the pooled analysis.

## Anastomotic time

A total of 6 studies [5, 9, 11–13, 15] were included in the analysis comparing the anastomotic time between continuous and interrupted suturing techniques, with a total of 988 patients (299 in the continuous group and 689 in the interrupted group). The heterogeneity between the included research results was substantial (I^2^ = 96%), so a random effects model was used for meta-analysis. The results showed that continuous suturing was associated with significantly shorter anastomotic time compared to interrupted suturing [MD = −13.06, 95% CI (−17.37, −8.75), *P* < 0.001, Fig. 3A]. The absolute meantime was **14.4** min in the continuous group compared to **25** min in the interrupted group.

Among the included studies, only Saxane et al. (2021) [11], and Seifert et al. (2022) [13] specified a size-based approach to selecting the suturing technique. In their protocol, a common hepatic duct diameter of 8 mm served as the decision threshold: interrupted suturing was used for ducts smaller than 8 mm, while continuous suturing was performed in ducts measuring 8 mm or larger. None of the other included studies reported a similar bile duct size cut-off for guiding the choice of technique.

Sensitivity analysis through the leave-one-out method demonstrated consistent findings, with mean differences ranging from −10.77 to −14.54 min, all maintaining statistical significance (P < 0.001). The removal of Saxane 2021 resulted in the reduction in heterogeneity from I^2^ = 96% to I^2^ = 90.8% (Supplementary Fig. 1).

## Costs

Totally 4 studies [9, 11, 13, 15] were included in the analysis comparing costs between continuous and interrupted suturing techniques, with a total of 808 patients (208 in the continuous group and 600 in the interrupted group). The heterogeneity between the included research results was substantial (I^2^ = 91%), so a random effects model was used for meta-analysis. The results showed that continuous suturing was associated with significantly lower costs compared to interrupted suturing [SMD = −4.89, 95% CI (−6.10, −3.67), P < 0.001, Fig. 3B]. The absolute mean costs were **$15.73** in the continuous group compared to **$105.30** in the interrupted group. Sensitivity analysis through the leave-one-out method demonstrated consistent findings, with standardized mean differences ranging from −4.69 to −5.38, all maintaining statistical significance (*P* < 0.001), the removal of Saxane 2021 resulted in a complete elimination of heterogeneity from I^2^ = 91% to I^2^ = 0%. (Supplementary Fig. 2).

## Post-operative outcomes

### Anastomotic stricture

Totally 7 studies [5, 9, 11–15] were included in the analysis comparing anastomotic stricture between continuous and interrupted suturing techniques, with a total of 1048 patients (337 in the continuous group and 711 in the interrupted group). The heterogeneity between the included research results was low (I^2^ = 0%), so a fixed effect model was used for meta-analysis. The results showed no significant difference in anastomotic stricture between continuous and interrupted suturing [RR = 1.24, 95% CI (0.68, 2.27), *P* = 0.481, Fig. 2A]. The absolute event rates were 16/337 (4.7%) in the continuous group compared to 29/711 (4.1%) in the interrupted group. Sensitivity analysis through the leave-one-out method demonstrated consistent findings, with risk ratios ranging from 0.94 to 1.60, all maintaining statistical non-significance (*P* > 0.05). (Supplementary Fig. 3).

## Bile leakage

Totally 7 studies [5, 9, 11–15] were included in the analysis comparing bile leakage between continuous and interrupted suturing techniques, with a total of 1159 patients (415 in the continuous group and 744 in the interrupted group).

The heterogeneity between the included research results was low (I^2^ = 0%), so a fixed effect model was used for meta-analysis. The results showed no significant difference in bile leakage rates between continuous and interrupted suturing [RR = 0.80, 95% CI (0.45, 1.43), *P* = 0.459]. The absolute event rates were 16/415 (3.9%) in the continuous group compared to 58/744 (7.8%) in the interrupted group. Sensitivity analysis through leave-one-out method demonstrated consistent findings, with risk ratios ranging from 0.78 to 0.98, all maintaining statistical non-significance (P > 0.05). As per (Supplementary Fig. 4).

## Cholangitis

Totally 3 studies [5, 11, 12] were included in the analysis comparing cholangitis rates between continuous and interrupted suturing techniques, with a total of 413 patients (237 in the continuous group and 176 in the interrupted group). The heterogeneity between the included research results was low (I^2^ = 0%), so a fixed effects model was used for meta-analysis. The results showed that there was no significant difference in cholangitis rates between the continuous and interrupted groups [RR = 0.89, 95% CI (0.57, 1.39), *P* = 0.600]. A total of 29 cholangitis events were reported in the continuous group compared to 30 events in the interrupted group. Sensitivity analysis through the leave-one-out method demonstrated consistent findings, with risk ratios ranging from 0.75 to 5.38, none reaching statistical significance. (Supplementary Fig. 5).

## Short-term morbidity

Totally 2 studies [5, 9], were included in the analysis comparing short-term morbidity between continuous and interrupted suturing techniques, with a total of 156 patients (74 in the continuous group and 82 in the interrupted group).

The heterogeneity between the included research results was low (I^2^ = 0%), so a fixed effect model was used for meta-analysis. The results showed no significant difference in short-term morbidity between continuous and interrupted suturing [RR = 0.71, 95% CI (0.48, 1.05), *P* = 0.089]. The absolute event rates were 21/74 (28.4%) in the continuous group compared to 30/82 (36.5%) in the interrupted group. Due to the limited number of studies, sensitivity analysis was not feasible, but the consistency of findings across different weight distributions supports the robustness of these results. Sensitivity analysis through the leave-one-out method demonstrated consistent findings, with risk ratios ranging from 0.71 to 0.97, all maintaining statistical non-significance (*P* > 0.05). As presented in (Supplementary Fig. 7).

## Length of hospital stay

**Fig. 4:**
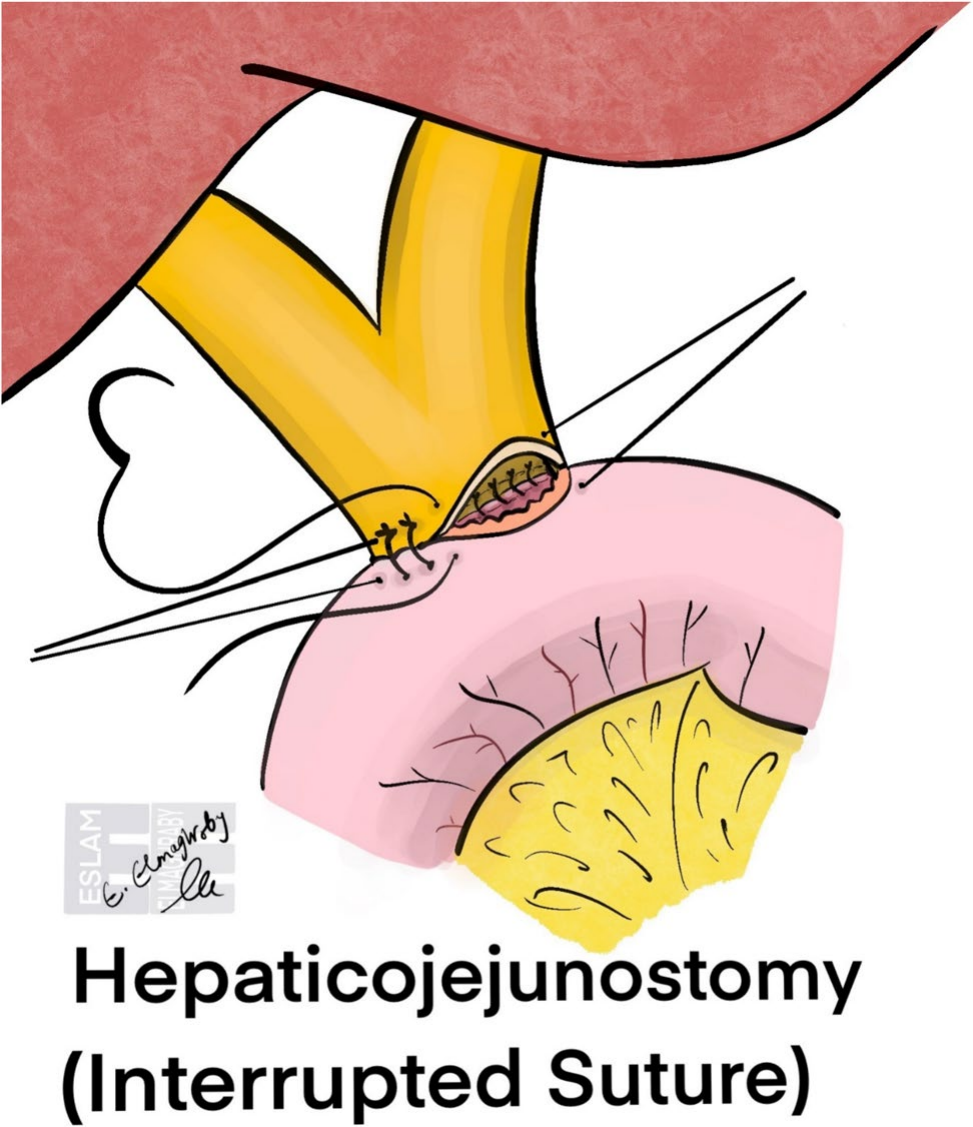
Schematic illustration of hepaticojejunostomy using interrupted sutures. Each stitch is placed individually through the bile duct and jejunal wall, tied separately to maintain tension and ensure watertight closure

Totally 2 studies [9, 13] were included in the analysis comparing the length of hospital stay between continuous and interrupted suturing techniques, with a total of 613 patients (112 in the continuous group and 501 in the interrupted group). The heterogeneity between the included research results was low (I^2^ = 0%), so a fixed effects model was used for meta-analysis. The results showed that there was no significant difference in the length of hospital stay between the continuous and interrupted groups [MD = 0.28 days, 95% CI (−0.23, 0.79), *P* = 0.284, Fig. 4]. The mean hospital stay was 6.34 days in the continuous group compared to 6.06 days in the interrupted group. Due to the limited number of studies, sensitivity analysis was not done. As presented in (Supplementary Fig. 6).

## Overall morbidity

Totally 4 studies [5, 9, 12, 13] were included in the analysis comparing overall morbidity between continuous and interrupted suturing techniques, with a total of 865 patients (269 in the continuous group and 596 in the interrupted group). The heterogeneity between the included research results was low (I^2^ = 0%), so a fixed effect model was used for meta-analysis. The results showed no significant difference in overall morbidity between continuous and interrupted suturing [RR = 0.84, 95% CI (0.66, 1.08), *P* = 0.181, Fig. 2C]. The absolute event rates were 60/269 (22.3%) in the continuous group compared to 221/596 (37.1%) in the interrupted group. Sensitivity analysis through leave-one-out method demonstrated consistent findings, with risk ratios ranging from 0.70 to 0.88, all maintaining statistical non-significance (*P* > 0.05). (Supplementary Fig. 8).

## Re-exploration rate

Totally 4 studies [5, 9, 12, 13] were included in the analysis comparing re-exploration rates between continuous and interrupted suturing techniques, with a total of 770 patients (195 in the continuous group and 575 in the interrupted group). The heterogeneity between the included research results was low (I^2^ = 0%), so a fixed effect model was used for meta-analysis. The results showed no significant difference in re-exploration rates between continuous and interrupted suturing [RR = 1.71, 95% CI (0.53, 5.51), *P* = 0.366]. The absolute event rates were 6/195 (3.1%) in the continuous group compared to 5/575 (0.9%) in the interrupted group. Sensitivity analysis through the leave-one-out method demonstrated consistent findings, with risk ratios ranging from 1.36 to 2.34, all maintaining statistical non-significance (*P* > 0.05). (Supplementary Fig. 9).

## Risk of bias results

The risk of bias assessment revealed varying quality across the included studies. Among the two randomized controlled trials, Brunner et al. [5] demonstrated low risk of bias across most domains, while Seifert et al. [13] showed some concerns regarding deviations from intended interventions, measurement of outcomes, and selective reporting (Supplementary Fig. 1). The six non-randomized studies generally exhibited more substantial methodological limitations. Four studies [12, 14] (Saxena 2021 and Yadav 2024) were rated as having serious risk of bias overall, primarily due to confounding and selection bias. Only Tatsuguchi et al. [9], which used alternate allocation rather than true randomization, achieved a moderate risk of bias rating (Supplementary Fig. 2). Notably, all non-randomized studies showed at least moderate risk of bias in outcome measurement due to lack of blinding. The summary graphs (Supplementary Figs. 3 and 4) highlight that methodological quality was substantially higher in randomized trials compared to non-randomized studies, with confounding emerging as the most problematic domain across the non-randomized evidence base. Detailed risk of bias and quality assessments for each study, using the respective tools, are presented in Fig. 3.

## GRADE assessment

Our GRADE assessment revealed low-certainty evidence that continuous suturing significantly reduces the hepaticojejunostomy time and costs compared to interrupted suturing, with limitations due to risk of bias and heterogeneity. For clinical outcomes including anastomotic stricture, bile leakage, morbidity, and cholangitis, we found mostly low-certainty evidence showing no significant differences between techniques. Only overall survival showed moderate-certainty evidence of no difference (RR 1.01), while re-exploration rate evidence was very low-certainty. These findings suggest that while continuous suturing offers procedural advantages, better quality evidence is needed to establish its clinical safety with greater certainty [24], as per Table 4.

**Table 4:** GRADE SUMMARY: continuous versus interrupted suture technique for hepaticojejunostomy. GRADE assessment of evidence quality. The certainty of evidence for each outcome was evaluated using the GRADE approach. The risk of bias was judged based on study design (RCTs and observational studies), inconsistency was assessed using I^2^ statistics, and the width of confidence intervals determined imprecision. Outcomes were rated for overall certainty and clinical importance. Continuous suture (CS) and interrupted suture (IS) groups were compared across multiple endpoints. CI = Confidence interval; MD = Mean difference; SMD = Standardized mean difference; RR = Risk ratio; RCT = Randomized controlled trial

OUTCOME	No. OF STUDIES	Study Design	Risk of Bias	Inconsistency	Indirectness	Imprecision	Other Considerations	Number of Patients in Each Group	Effect (95% CI)	CERTAINTY	IMPORTANCE
Anastomotic Time	6	2 RCTs, 4 Observational	Serious (2 RCTs: low-some concerns, 4 studies: serious)	High (I^2^ = 96%)	None	None	None	299 CS/689 IS	MD −13.06 min (−17.37 to −8.75); P < 0.001	Low (⨁⨁◯◯)	High (★★★★☆)
Costs	4	1 RCT, 3 Observational	Serious (1 RCT: low, 3 studies: serious)	High (I^2^ = 91%)	None	None	None	208 CS/600 IS	SMD −4.89 (−6.10 to −3.67); P < 0.001	Low (⨁⨁◯◯)	Moderate (★★★☆☆)
Anastomotic Stricture	7	2 RCTs, 5 Observational	Serious (2 RCTs: low-some concerns, 5 studies: serious-moderate)	Low (I^2^ = 0%)	None	Serious (wide CI)	None	337 CS/711 IS	RR 1.24 (0.68 to 2.27); *P* = 0.481	Low (⨁⨁◯◯)	High (★★★★☆)
Bile Leakage	7	2 RCTs, 5 Observational	Serious (2 RCTs: low-some concerns, 5 studies: serious-moderate)	Low (I^2^ = 0%)	None	Serious (wide CI)	None	422 CS/768 IS	RR 0.85 (0.58 to 1.26); *P* = 0.421	Low (⨁⨁◯◯)	High (★★★★☆)
Short-term Morbidity	2	1 RCT, 1 Observational	Serious (1 RCT: some concerns, 1 studies: serious)	Low (I^2^ = 0%)	None	Serious (wide CI)	None	81 CS/106 IS	RR 0.78 (0.59 to 1.03); P = 0.078	Low (⨁⨁◯◯)	High (★★★★☆)
Overall Morbidity	4	1 RCT, 3 Observational	Serious (1 RCT: some concerns, 3 studies: serious)	Low (I^2^ = 0%)	None	Serious (wide CI)	None	269 CS/596 IS	RR 0.84 (0.66 to 1.08); *P* = 0.181	Low (⨁⨁◯◯)	High (★★★★☆)
Cholangitis	3	1 RCT, 2 Observational	Serious (1 RCT: some concerns, 2 studies: serious)	Low (I^2^ = 0%)	None	Serious (wide CI)	None	237 CS/176 IS	RR 0.89 (0.57 to 1.39); *P* = 0.600	Low (⨁⨁◯◯)	High (★★★★☆)
Length of Hospital Stay	2	1 RCT, 1 Observational	Serious (1 RCT: low, 1 study: serious)	Low (I^2^ = 0%)	None	Serious (wide CI)	None	112 CS/501 IS	MD 0.28 days (−0.23 to 0.79); *P* = 0.284	Low (⨁⨁◯◯)	Moderate (★★★☆☆)
Re-Exploration Rate	4	1 RCT, 3 Observational	Serious (1 RCT: low, 3 studies: serious)	Low (I^2^ = 0%)	None	Very serious (very wide CI)	None	195 CS/575 IS	RR 1.71 (0.53 to 5.51); *P* = 0.366	Very Low (⨁◯◯◯)	High (★★★★☆)

*CS* Continuous suture; *IS* Interrupted suture; *RCT* Randomized controlled trial; *MD* Mean difference; *SMD* Standardized mean difference; *RR* Risk ratio; *CI* Confidence interval

## Discussion

**Fig. 5:**
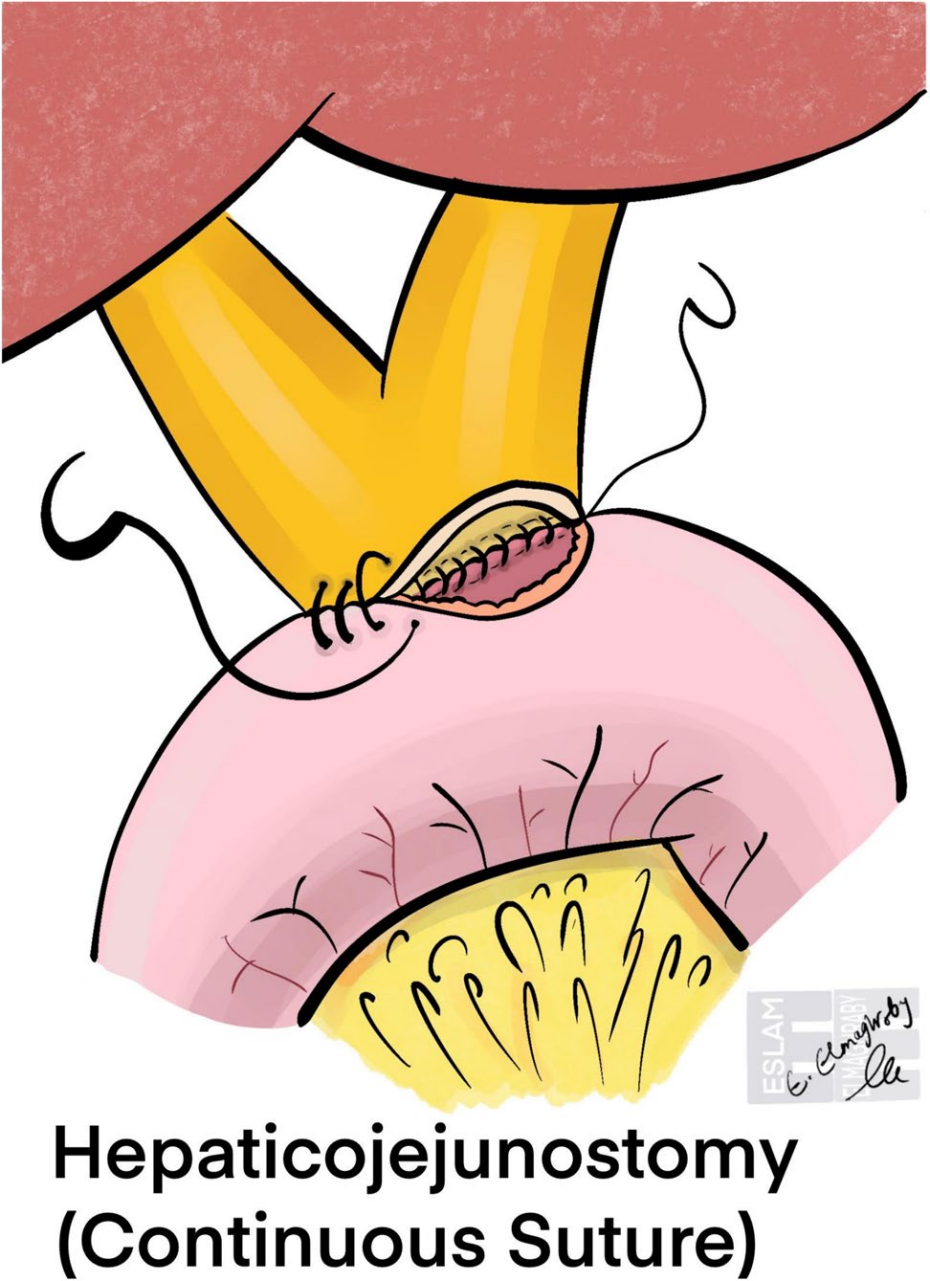
Schematic illustration of hepaticojejunostomy using continuous sutures. A single running suture is used to approximate the bile duct and jejunal mucosa, providing uniform tension and potentially reduced operative time

Hepaticojejunostomy (HJ) is a key reconstructive step in patients with upper GIT surgery, and HBP surgery, with implications for both immediate surgical outcomes and long-term patient prognosis. The choice between interrupted (Fig. 4) and continuous suturing (Fig. 5) techniques remains controversial, as each method carries theoretical advantages and limitations [8].

This meta-analysis systematically compared operative efficiency (anastomotic time and cost) and postoperative outcomes (bile leakage, anastomotic stricture, morbidity, cholangitis, hospital stay, and re-exploration rates) between the two techniques. By consolidating available data, we sought to provide evidence-based recommendations to optimize surgical practice.

## Anastomotic time and surgical cost

Our meta-analysis supports the notion that continuous suturing is associated with significantly shorter anastomotic time and lower surgical cost compared to interrupted suturing, thereby improving overall operative efficiency.

The time efficiency of continuous suturing aligns with findings from prior studies [9, 10, 12], which highlight its streamlined technique, fewer suture placements, and continuous tension distribution, which allow for faster completion without compromising integrity, especially for wide CHD (common hepatic duct) [33]. However, some surgeons argue that interrupted suturing may offer better precision, particularly in complex cases or when dealing with narrow bile ducts [34]. Notably, despite the statistical significance of this time reduction, its clinical relevance remains debatable, as PD is a 4–6-h procedure, making a 10-min reduction in anastomotic time unlikely to influence overall operative efficiency or patient outcomes [35].

Continuous suturing was also associated with a substantial reduction in operative costs. This is plausibly attributed to the decreased use of suture material and the shorter anastomotic time, which indirectly reduces anesthesia time and operating room occupancy. However, cost-effectiveness alone should not dictate surgical technique, particularly if clinical outcomes are compromised [36]. While some studies advocate for continuous suturing as a cost-effective alternative, others emphasize that the economic advantage is marginal when compared to the overall costs of PD and its potential postoperative complications [37]. Further, it remains unclear whether these savings translate into meaningful reductions in hospital expenses or patient billing.

A major contributor to the heterogeneity in anastomotic time and cost was the study by Saxane et al. (2021) [11], which differed significantly from other studies in both surgical context and intraoperative technique. Conducted in a high-volume Indian center, the study included the largest patient cohort, utilized different suture materials and techniques, and reported markedly higher suture counts and anastomotic durations in the interrupted group. These intraoperative differences, coupled with a healthcare system operating under different economic constraints, likely contributed to the divergence in outcome measures. While sensitivity analyses confirmed that these differences did not materially change the overall conclusions, the influence of such outlier data underscores the need for more standardized reporting in future comparative studies.

## Postoperative outcomes

Across all included studies, no significant differences were observed between suturing techniques in terms of anastomotic stricture, bile leakage, overall morbidity, cholangitis, re-exploration rates, or length of hospital stay. These findings suggest that when performed with surgical precision, both techniques are safe and effective.

## Anastomotic stricture

Anastomotic strictures are a serious long-term complication of HJ, potentially leading to biliary obstruction, cholangitis, and the need for re-intervention [38]. Our analysis showed no significant difference between continuous and interrupted suturing in terms of stricture rates. Previous studies have debated whether continuous suturing compromises anastomotic pliability, increasing fibrosis and strictures over time [39–41]. However, our results do not support this concern, indicating that when performed with meticulous technique, both suturing methods provide equivalent long-term patency. Furthermore, the low heterogeneity (I^2^ = 0%) strengthens the reliability of this finding.

## Bile leakage

Bile leakage remains a major early postoperative complication of HJ, often leading to infections, peritonitis, sepsis, prolonged hospital stays, and potential re-interventions [42]. Our analysis found no significant difference in bile leakage rates between both groups.

Some studies suggest that interrupted suturing allows better microvascular perfusion at the anastomotic site, potentially reducing the risk of ischemia-related leakage [43–46]. However, others argue that continuous suturing provides better watertight closure, particularly in wide bile ducts, leading to reduced biliary extravasation [47, 48]. The absence of a significant difference in our meta-analysis suggests that technical proficiency and patient factors may be more critical determinants of bile leakage than the choice of suturing technique.

**Fig. 6:**
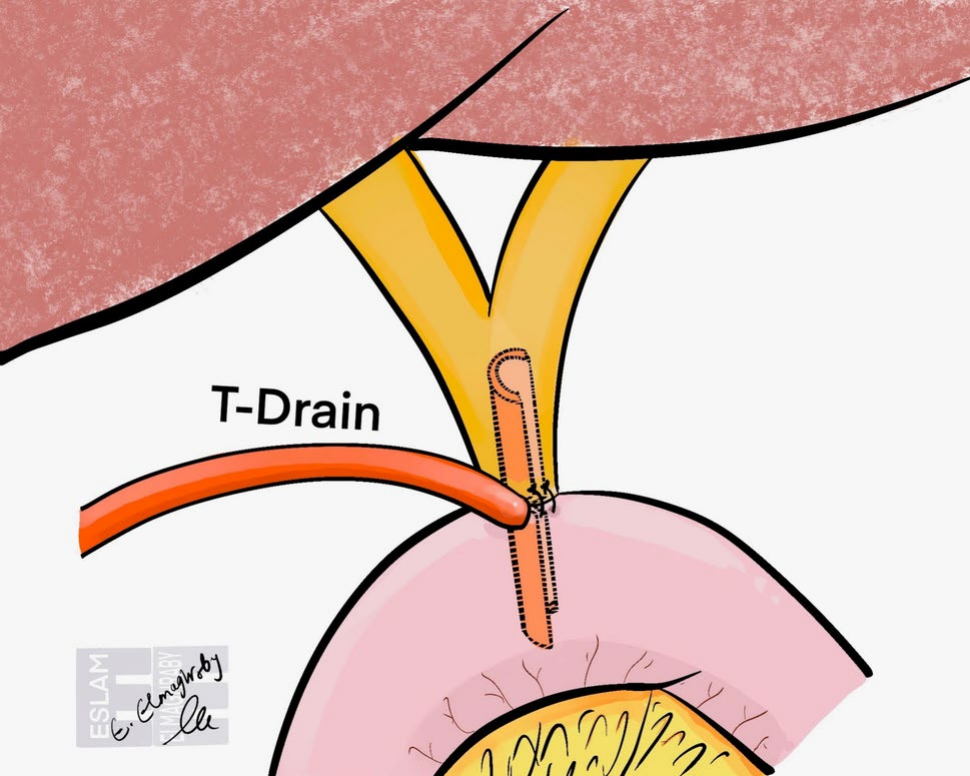
Schematic view of a hepaticojejunostomy anastomosis augmented with a T-drain. The drain passes through the anastomosis into the jejunum, used to decompress the biliary system and reduce the severity of bile leakage in high-risk cases

For difficult hepaticojejunostomies, particularly in cases with small hepatic ducts, alternative strategies have been explored to minimize bile leakage. Herzog et al. [49] demonstrated that T-tube augmentation at the anastomotic site (Fig. 6) does not prevent biliary leakage but does reduce its severity, resulting in fewer reoperations [49]. This finding highlights the importance of adjunctive techniques in high-risk anastomoses and suggests that while T-tubes may not eliminate leakage, they can mitigate its clinical impact. These insights further emphasize that the choice of suturing technique alone may not be the primary determinant of anastomotic integrity, and tailored approaches should be considered based on duct size, patient factors, and surgeon expertise.

## Short-term morbidity & overall morbidity

No significant differences were observed between continuous and interrupted suturing in terms of short-term morbidity or overall morbidity. These findings suggest that both techniques are equally safe, provided they are executed with surgical expertise.

While prior studies have indicated theoretical advantages of interrupted suturing in minimizing ischemic stress at the anastomotic site [43–46], our results indicate that this does not translate into measurable reductions in morbidity. The lack of heterogeneity (I^2^ = 0%) further strengthens the robustness of this conclusion.

## Cholangitis

Postoperative cholangitis, a potential consequence of anastomotic dysfunction or strictures [50], showed no significant difference between both groups. These findings support the notion that both suturing techniques maintain adequate anastomotic integrity and do not predispose patients to increased infection risk.

A notable limitation is the lack of standardized definitions for cholangitis across the included studies. None of the studies referenced established diagnostic criteria, such as the Tokyo Guidelines, leading to potential heterogeneity in how this outcome was diagnosed and reported. Future trials should adopt standardized criteria to ensure consistency and reliability in reporting biliary complications.

## Length of hospital stay

Our meta-analysis found no significant difference in length of hospital stay. The mean hospital stay was equal (about 6 days) in both groups. This finding reinforces the idea that early recovery and discharge are more influenced by overall surgical outcomes, patient comorbidities, and postoperative management rather than the suturing technique alone [51, 52].

## Re-exploration rate

Similarly, the two groups had no significant differences in re-exploration rates. Given that re-explorations are often necessitated by major complications (e.g., bile leaks, abscesses, or bleeding) [53–55], our findings suggest that both suturing techniques are equally reliable in maintaining anastomotic integrity.

## Suture material considerations

An important yet often overlooked factor in hepaticojejunostomy is the choice of suture material. Across the included studies, various materials and calibers were used; however, most authors did not explicitly justify their selection, suggesting that the choice may reflect institutional standards or surgeon preference rather than evidence-based decision-making. Saxane et al. [11] predominantly used Vicryl, while Natsume et al. [12], Seifert et al. [13], and most hospitals in Germany, as reported by Brunner et al. [5, 10], preferred monofilament absorbable sutures such as PDS 5.0. Interestingly, the Brunner survey of 77 German hospitals found that university hospitals tended to use significantly finer suture calibers (e.g., PDS 5.0 or 6.0), with *P* < 0.001. Tatsuguchi et al. [9] reported using PDS for interrupted suturing and Vicryl 4.0 for continuous suturing, whereas Sabra et al. [14] used Vicryl 5.0, and Yadav et al. [15] employed PDS 4.0.

Given that suture characteristics such as absorbability, elasticity, and tissue reactivity can influence healing and anastomotic integrity, future studies should address the impact of suture material on outcomes more systematically.

## Clinical implications

These results suggest that continuous suturing may offer practical benefits in terms of efficiency and cost without compromising clinical outcomes. Interrupted suturing remains the most commonly used approach, and surgeons with proven success should not alter their technique based solely on these differences. For experienced surgeons, particularly in high-volume centers, adopting a continuous approach could streamline the procedure, especially in robotic or minimally invasive procedures, and may be appropriate when the overall procedure is expected to remain within a limited operative timeframe (such as HJ for choledochal cysts, palliative bypass procedures, or hemodynamically unstable patients—especially when no major resection is planned). However, in anatomically challenging cases—such as those involving narrow bile ducts or extensive inflammation—interrupted suturing may still be preferred for its precision and adaptability.

## Limitations

Although the strength of our meta-analysis lies in its comprehensive comparison of continuous versus interrupted suturing in hepaticojejunostomy across diverse studies, several limitations must be acknowledged when interpreting the findings.

First, while our results demonstrate a statistically significant reduction in anastomotic time and material costs with continuous suturing, the clinical relevance of these differences may vary depending on the surgical context. In complex hepatopancreatobiliary (HPB) procedures, where operations inherently involve extended durations and often necessitate prolonged intensive care monitoring, a time saving of approximately 10 min may be of limited practical value. This underscores the importance of contextualizing technical efficiency within the broader operative and postoperative framework.

Second, significant heterogeneity was observed in the pooled estimates for anastomotic time and cost (I^2^ = 96% and I^2^ = 91%, respectively), likely reflecting variability in surgical expertise, patient complexity, and institutional protocols across the included studies. The shortage of randomized controlled trials further limits the robustness of our conclusions, as the majority of included studies were observational in nature, introducing potential selection bias.

Third, patient selection criteria were inconsistently reported, with considerable variation in bile duct diameter, comorbidities, and intraoperative circumstances—all of which may influence both surgical difficulty and outcomes. Specifically, the diameter of the bile duct is a known determinant of anastomotic complexity and duration; however, with the exception of two studies (Saxane et al. 2021 [11]; Seifert et al. [13]), which used an 8 mm threshold, most studies failed to report this parameter. Consequently, we were unable to perform subgroup analyses based on duct size or identify a specific diameter above which continuous suturing may offer greater benefit. This highlights the need for future studies to report individual patient data and stratify outcomes by relevant anatomical variables.

Finally, follow-up durations varied substantially between studies and were often insufficiently reported, limiting the ability to assess long-term complications such as anastomotic stricture formation. Moreover, survival and follow-up parameters were not clearly defined or uniformly reported across the included studies. In light of this inconsistency and the substantial heterogeneity of surgical indications within the analyzed cohorts, we have removed the survival analysis component from the current manuscript, as its interpretive value would have been limited and potentially misleading.

## Conclusion

Continuous suturing in hepaticojejunostomy significantly reduces anastomotic time by approximately 10 min and lowers material costs by around $90, without increasing postoperative morbidity. Interrupted suturing remains the most commonly employed technique, particularly in high-volume centers and survey-based studies. Consequently, the choice of suturing method should be based on surgeon expertise, institutional protocols, and patient-specific considerations. Further well-designed randomized controlled trials with standardized outcome definitions and stratified patient cohorts are warranted to confirm these results and support the development of evidence-based surgical protocols in HPB surgery.

## Supplementary Information

Below is the link to the electronic supplementary material.

Supplementary file1 (DOCX 22 KB)

Supplementary file2 (DOCX 15 KB)

Supplementary file3 (XLSX 210 KB)

Supplementary file4 (DOCX 385 KB)

## Data Availability

No datasets were generated or analysed during the current study.

## Abbreviations

**CHD**: Common hepatic duct
**GIT**: Gastrointestinal tract
**HBP**: Hepaticobiliarypancreatic (HBP) surgery
**HJ**: Hepaticojejunostomy
**PD**: Pancreaticoduodenectomy
**RCTs**: Randomized controlled trials
