# Supplementary material for: Continuous vs. interrupted suturing in hepaticojejunostomy: a comprehensive systematic review and meta-analysis
Source: Langenbecks Arch Surg. 2025 Jul 4;410(1):214. doi: 10.1007/s00423-025-03756-y (PMC12227507; doi:10.1007/s00423-025-03756-y)
Supplement: Supplementary file 1 — Supplementary file1 (DOCX 22 KB) [file 423_2025_3756_MOESM1_ESM.docx]

| Year | Author | Title | Research type | DOI | Link |
| --- | --- | --- | --- | --- | --- |
| 2022 | M. Brunner, H. Golcher, C. Krautz, S. Kersting, G. F. Weber and R. Grutzmann | Continuous or interrupted suture technique for hepaticojejunostomy during pancreatoduodenectomy (HEKTIK trial): study protocol of a randomized controlled multicenter trial | Journal article | 10.1186/s13063-022-06427-1 | <https://www.cochranelibrary.com/central/doi/10.1002/central/CN-02402519/full> |
| 2019 | ChiCtr | Continuous suture Versus interrupted suture in Hepaticojejunostomy After Pancreaticoduodenectomy: a Randomized Controlled Trial | Trial registry record |  | <https://www.cochranelibrary.com/central/doi/10.1002/central/CN-01949250/full> |
| 2021 | Ctri | TO COMPARE DIFFERENT SUTURING TECHNIQUES IN HEPATICOJEJUNOSTOMY - A RANDOMIZED CONTROLLED TRIAL | Trial registry record |  | <https://www.cochranelibrary.com/central/doi/10.1002/central/CN-02282244/full> |
| 2021 | Drks | Continuous versus interrupted suture technique for hepaticojejunostomy (HEKTIK trial) | Trial registry record |  | <https://www.cochranelibrary.com/central/doi/10.1002/central/CN-02240957/full> |
| 2005 | D. Kelemen and Ö. Horváth | Experiences with single-layer biliodigestive anastomosis |  |  | <Go to ISI>://WOS:000229759000008 |
| 2018 | Y. Liu, M. Diao and L. Li | Using laparoscopic double hemicircumferential running single-layer suture in hepaticojejunostomy for choledochal cysts in children | Article | 10.1016/j.jpedsurg.2017.09.015 | <https://www.scopus.com/inward/record.uri?eid=2-s2.0-85044277583&doi=10.1016%2fj.jpedsurg.2017.09.015&partnerID=40&md5=d559a7d289f84a16e0a88edb58659b63> |
| 2005 | S. Moriura, I. Kobayashi, K. Hattori, T. Oshiro, M. Kawahara and T. Matsumoto | Continuous vertical hemimattress suture for biliary-enteric anastomosis |  | 10.1007/s00534-005-1008-x |  |
| 2021 | Y. Nagakawa, S. Kozono, C. Takishita, H. Osakabe, H. Nishino, N. Nakagawa, K. Suzuki, Y. Hayashi, T. Ishizaki, K. Katsumata and A. Tsuchida | Incidence of anastomotic stricture after hepaticojejunostomy with continuous sutures in patients who underwent laparoscopic pancreaticoduodenectomy |  | 10.1007/s00595-020-02223-z |  |
| 2022 | N. Napoli, E. F. Kauffmann, R. Caputo, M. Ginesini, F. Asta, C. Gianfaldoni, G. Amorese, F. Vistoli and U. Boggi | Outcomes of double-layer continuous suture hepaticojejunostomy in pancreatoduodenectomy and total pancreatectomy |  | 10.1016/j.hpb.2022.05.005 |  |
