# Supplementary material for: Continuous vs. interrupted suturing in hepaticojejunostomy: a comprehensive systematic review and meta-analysis
Source: Langenbecks Arch Surg. 2025 Jul 4;410(1):214. doi: 10.1007/s00423-025-03756-y (PMC12227507; doi:10.1007/s00423-025-03756-y)
Supplement: Supplementary file 2 — Supplementary file2 (DOCX 15 KB) [file 423_2025_3756_MOESM2_ESM.docx]

**"Continuous vs. Interrupted Suturing in Hepaticojejunostomy After Pancreaticoduodenectomy: A comprehensive Systematic Review and Meta-Analysis"**

**Search strategy:**

1. (Sutures OR suture)
2. (Hepaticojejunostomy OR “Biliary-enteric anastomosis” OR Choledochojejunostomy OR “Hepatic duct jejunostomy” OR “Biliary reconstruction with jejunal loop” OR “Jejunal interposition for biliary bypass”)
3. 1 AND 2

**Pubmed: 173 results**

((suture[Title/Abstract]) OR (sutures[Title/Abstract])) AND ((((Hepaticojejunostomy[Title/Abstract]) OR ("Biliary-enteric anastomosis"[Title/Abstract])) OR (Choledochojejunostomy[Title/Abstract])) OR ("Hepatic duct jejunostomy"[Title/Abstract]))

**Scopus: 171**

TITLE-ABS ( sutures OR suture ) AND TITLE-ABS ( hepaticojejunostomy OR "Biliary-enteric anastomosis" OR choledochojejunostomy OR "Hepatic duct jejunostomy" OR "Biliary reconstruction with jejunal loop" OR "Jejunal interposition for biliary bypass" )

**Web of science: 203**

TS=(( sutures OR suture ) AND ( hepaticojejunostomy OR "Biliary-enteric anastomosis" OR choledochojejunostomy OR "Hepatic duct jejunostomy" OR "Biliary reconstruction with jejunal loop" OR "jejunal interposition for biliary bypass"))

**Cochrane: 23**

( sutures OR suture ) AND ( hepaticojejunostomy OR "Biliary-enteric anastomosis" OR choledochojejunostomy OR "Hepatic duct jejunostomy" OR "Biliary reconstruction with jejunal loop" OR "Jejunal interposition for biliary bypass" )

**Embase:**

Search was not done/no access

Exported to endnote and duplicates removed

Total **570**

After removal of duplicates total number: **341**
