# Supplementary material for: Continuous vs. interrupted suturing in hepaticojejunostomy: a comprehensive systematic review and meta-analysis
Source: Langenbecks Arch Surg. 2025 Jul 4;410(1):214. doi: 10.1007/s00423-025-03756-y (PMC12227507; doi:10.1007/s00423-025-03756-y)
Supplement: Supplementary file 4 — Supplementary file4 (DOCX 385 KB) [file 423_2025_3756_MOESM4_ESM.docx]

# Anastomotic Time

# (Supplementary Fig. 1)

## Meta−analysis of Anastomotic Time: Continuous vs. Interrupted Sutures

**Study**

Tatsuguishi 2018

Saxane 2021

Seifert 2022

Sabra 2024

Yadav 2024

Brunner 2024

**Overall**

**Weight**

20.2%

7.4%

46.4%

11.3%

3.2%

11.5%

**100%**

**Continuous Mean (SD)**

16.20 (5.00)

21.40 (11.20)

12.79 (1.83)

15.30 (4.10)

16.20 (3.10)

11.60 (3.22)

**14.37**

**N**

80

88

41

24

16

50

**299**

**Interrupted Mean (SD)**

27.00 (6.60)

44.60 (20.30)

22.27 (3.35)

23.50 (5.20)

38.60 (9.20)

17.45 (8.00)

**24.99**

**N**

81

468

39

33

18

50

**689**

**Mean Difference [95% CI]**

−10.80 [−12.61, −8.99]

−23.20 [−26.18, −20.22]

−9.48 [−10.67, −8.29]

−8.20 [−10.62, −5.78]

−22.40 [−26.91, −17.89]

−5.85 [−8.24, −3.46]

**−13.06 [−17.37, −8.75]**

Heterogeneity: Tau² = 27.09; Chi² = 117.50, df = 5 (P = 0.000); I² = 96% Test for overall effect: Z = −5.94 (P = 0.000)

−40 −30 −20 −10 0 10 20

Mean Difference (Minutes)

Favors Continuous Favors Interrupted

## Leave−One−Out Analysis of Anastomotic Time Continuous vs. Interrupted Suturing

### Analysis None (Overall)

Removed: Tatsuguishi 2018

Removed: Saxane 2021

Removed: Seifert 2022

Removed: Sabra 2024

Removed: Yadav 2024

Removed: Brunner 2024

### Mean Difference [95% CI]

**−13.06 [−17.37, −8.75]**

−13.62 [−19.30, −7.94]

−10.77 [−13.88, −7.66]

−13.92 [−20.00, −7.85]

−14.09 [−19.23, −8.95]

−11.41 [−15.64, −7.17]

−14.54 [−19.40, −9.68]

### P−value 0.000

0.000

0.000

0.000

0.000

0.000

0.000

**I² 95.7%**

96.6%

90.8%

96.4%

96.5%

95.6%

96.0%

−40 −30 −20 −10 0 10 20

Mean Difference (Random−Effects Model)

# Cost

# (Supplementary Fig. 2)

## Cost outcome between continuous and interrupted suturing

**Study**

Tatsuguishi 2018

16.3%

11.70 (2.90)

80

144.70 (34.60)

81

−5.40 [−6.07, −4.73]

Saxane 2021

74.4%

17.42 (2.91)

88

104.18 (26.08)

468

−3.62 [−3.93, −3.31]

Sabra 2024

6.1%

5.17 (0.00)

24

33.00 (7.04)

33

−5.18 [−6.27, −4.10]

Yadav 2024

3.2%

17.15 (3.61)

16

68.94 (12.09)

18

−5.66 [−7.16, −4.15]

**Overall**

**100%**

**15.73**

**208**

**105.30**

**600**

**−4.89 [−6.10, −3.67]**

**Weight**

**Continuous Mean (SD) N**

**Interrupted Mean (SD) N**

**SMD [95% CI]**

Favors Continuous

−8 −6 −4 −2 0 2

Standardized Mean Difference (SMD)

Heterogeneity: Tau² = 1.30; Chi² = 31.69, df = 3 (P = 0.000); I² = 91% Test for overall effect: Z = −7.90 (P = 0.000)

## Leave−One−Out Analysis of Cost

### Study

**None (Overall)**

Removed: Tatsuguishi 2018

Removed: Saxane 2021

Removed: Sabra 2024

Removed: Yadav 2024

**SMD [95% CI]**

### −4.89 [−6.10, −3.67]

−4.69 [−6.09, −3.29]

−5.38 [−5.91, −4.85]

−4.80 [−6.28, −3.33]

−4.69 [−6.05, −3.33]

### P−value 0.000

0.000

0.000

0.000

0.000

**I² 90.5%**

85.0%

0.0%

92.7%

92.7%

−6 −5 −4 −3 −2 −1 0 1

Standardized Mean Difference (SMD)

Favors Continuous

# Anastomotic Stricture

# (Supplementary Fig. 3)

## Meta−analysis of Anastomotic Stricture: Continuous vs. Interrupted Sutures (Fixed Effect Model, * 0.5 Continuity Correction for Zero Events)

**Study**

Tatsuguishi 2018

Saxane 2021

Natsume 2021 Seifert 2022* Sabra 2024* Yadav 2024* Brunner 2024*

**Fixed effect model**

**Weight**

41.9%

38.3%

9.1%

2.9%

2.4%

2.7%

2.8%

**100%**

**Continuous Events/Total**

5/80

4/85

7/41

0/41

0/24

0/16

0/50

**16/337**

**Interrupted Events/Total**

7/81

21/455

1/35

0/39

0/33

0/18

0/50

**29/711**

**Risk Ratio [95% CI]**

0.74 [0.26, 2.14]

1.12 [0.41, 3.00]

4.28 [0.79, 23.29]

0.95 [0.02, 46.82]

1.37 [0.03, 66.55]

1.12 [0.02, 53.40]

1.00 [0.02, 49.43]

**1.24 [0.68, 2.27]**

Heterogeneity: Chi² = 2.99, df = 6 (P = 0.810); I² = 0% Test for overall effect: Z = 0.70 (P = 0.481)

0.10 0.20 0.50 1.0 2.0 5.00 10.00

Risk Ratio

Favors Continuous Favors Interrupted

## Leave−One−Out Analysis of Anastomotic Stricture (Fixed Effect Model, 0.5 Continuity Correction for Zero Events)

### Analysis None (Overall)

Removed: Tatsuguishi 2018

Removed: Natsume 2021

Removed: Seifert 2022

Removed: Sabra 2024

Removed: Yadav 2024

Removed: Brunner 2024

### Risk Ratio [95% CI]

**1.24 [0.68, 2.27]**

1.60 [0.75, 3.41]

0.94 [0.48, 1.84]

1.25 [0.68, 2.31]

1.24 [0.67, 2.29]

1.25 [0.68, 2.30]

1.25 [0.68, 2.30]

### P−value 0.481

0.220

0.855

0.473

0.491

0.481

0.475

**I² 0.0%**

0.0%

0.0%

0.0%

0.0%

0.0%

0.0%

−2.30258509299405 −0.693147180559945 0 0.693147180559945 1.6094379124341

Risk Ratio (Mantel−Haenszel Fixed−Effect)

# Bile Leakage

# (Supplementary Fig.4)

**
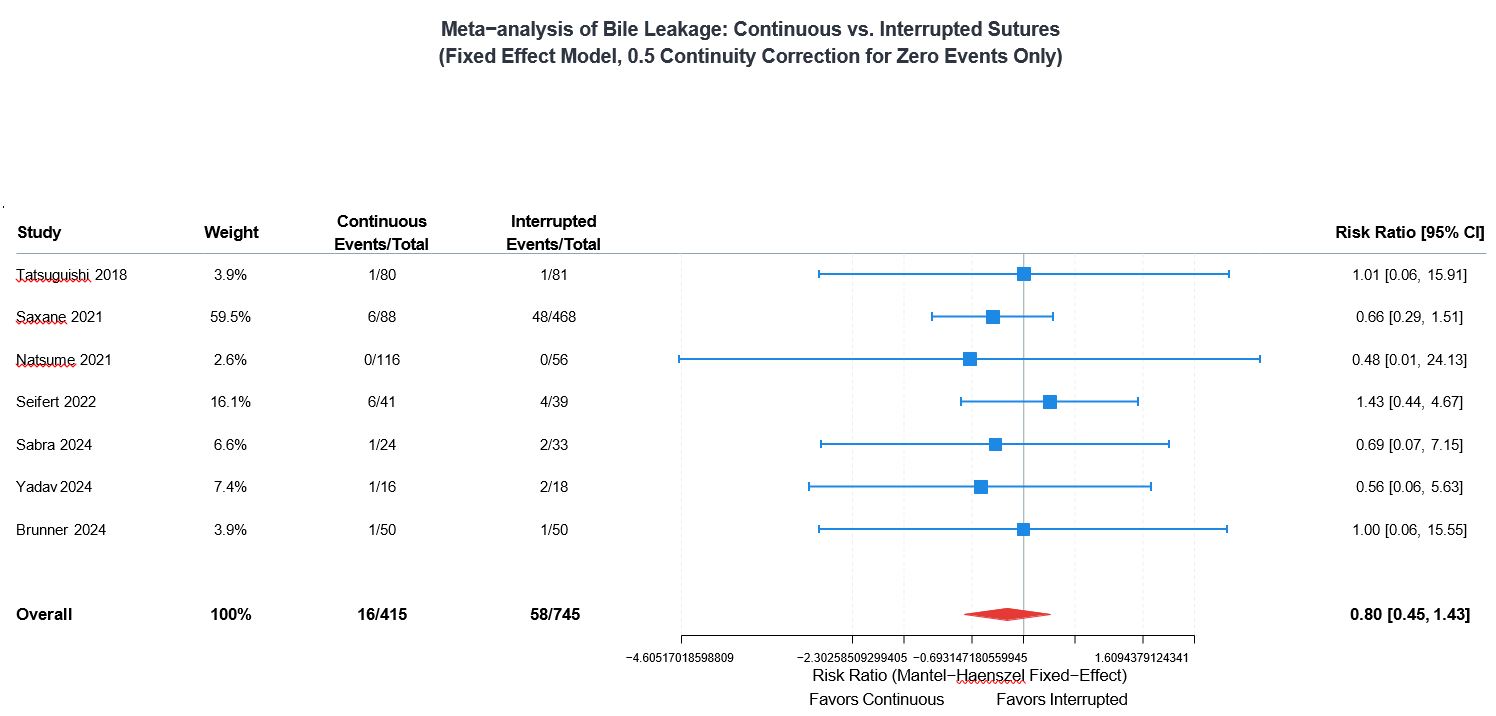
**

**
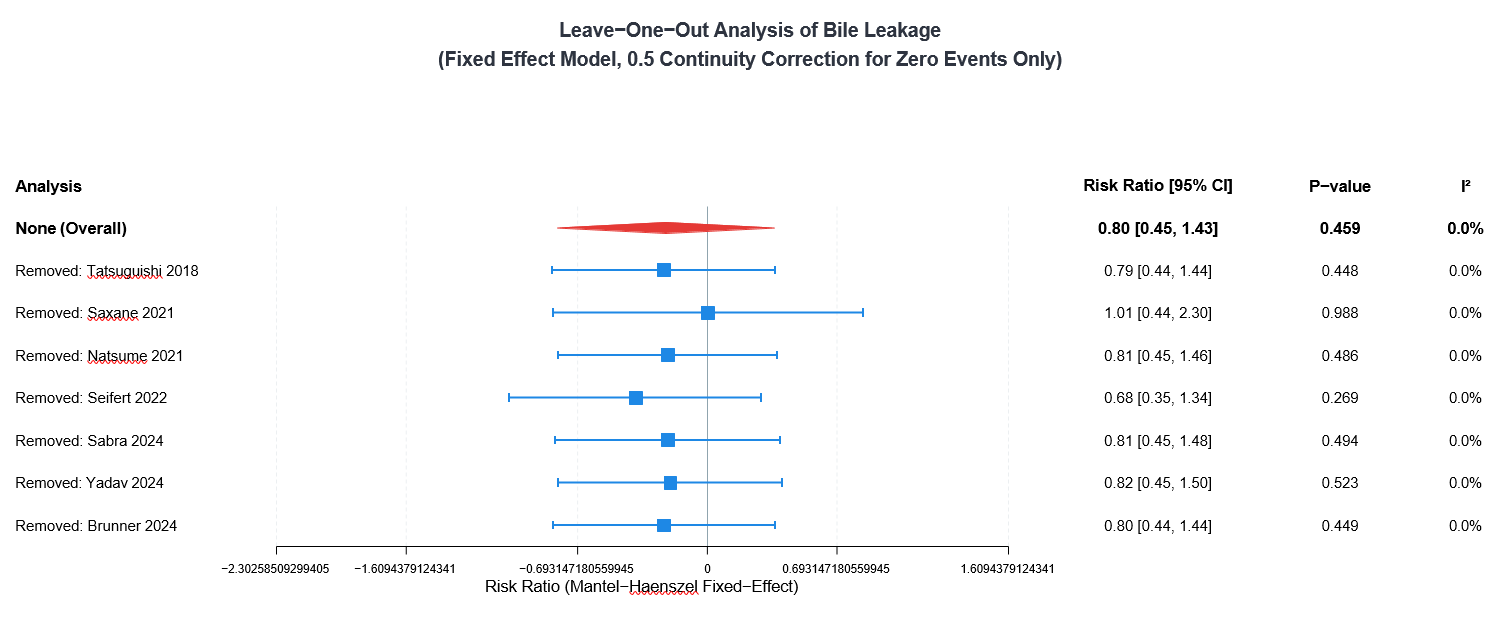
**

# Cholangitis

# (Supplementary Fig. 5)

## Meta−analysis of Cholangitis: Continuous vs. Interrupted Sutures (0.5 Continuity Correction for Zero Events Only)

**Study**

Tatsuguishi 2018

96.2%

21/80

30/81

0.71 [0.45, 1.13]

Natsume 2021

2.2%

7/116

0/56

7.27 [0.42, 125.14]

Seifert 2022

1.7%

1/41

0/39

2.86 [0.12, 68.04]

**Overall**

**100%**

**29/237**

**30/176**

**0.89 [0.57, 1.39]**

**Weight**

**Continuous Events/Total**

**Interrupted Events/Total**

**Risk Ratio [95% CI]**

Heterogeneity: Chi² = 3.17, df = 2 (P = 0.205); I² = 0% Test for overall effect: Z = −0.52 (P = 0.600)

−4.60517018598809 −1.6094379124341 0 1.6094379124341

Risk Ratio (Mantel−Haenszel Fixed−Effect) Favors Continuous Favors Interrupted

## Leave−One−Out Analysis of Cholangitis (0.5 Continuity Correction for Zero Events Only)

### Analysis None (Overall)

Removed: Tatsuguishi 2018

Removed: Natsume 2021

Removed: Seifert 2022

### Risk Ratio [95% CI]

**0.89 [0.57, 1.39]**

5.38 [0.64, 45.31]

0.75 [0.47, 1.18]

0.85 [0.54, 1.34]

### P−value 0.600

0.121

0.207

0.495

**I² 0.4%**

0.0%

0.0%

0.6%

−2.30258509299405−0.693147180559945 1.6094379124341

Risk Ratio (Mantel−Haenszel Fixed−Effect)

# Hospital Stays

# (Supplementary Fig.6)

## Meta−analysis of Hospital Stay: Continuous vs. Interrupted Sutures

**Study**

**Weight**

**Continuous Mean (SD) N**

**Interrupted Mean (SD) N**

**Mean Difference [95% CI]**

Saxane 2021

1.6%

20.75 (17.26)

88

21.75 (19.41)

468

−1.00 [−5.01, 3.01]

Sabra 2024

98.4%

6.10 (1.10)

24

5.80 (0.78)

33

0.30 [−0.21, 0.81]

**Overall**

**100%**

**6.34**

**112**

**6.06**

**501**

**0.28 [−0.23, 0.79]**

Heterogeneity: Chi² = 0.40, df = 1 (P = 0.529); I² = 0% Test for overall effect: Z = 1.07 (P = 0.284)

−4 −3 −2 −1 0 1 2 3 4

Mean Difference (Days)

Favors Continuous Favors Interrupted

# Short-term Morbidity

# (Supplementary Fig.7)

**
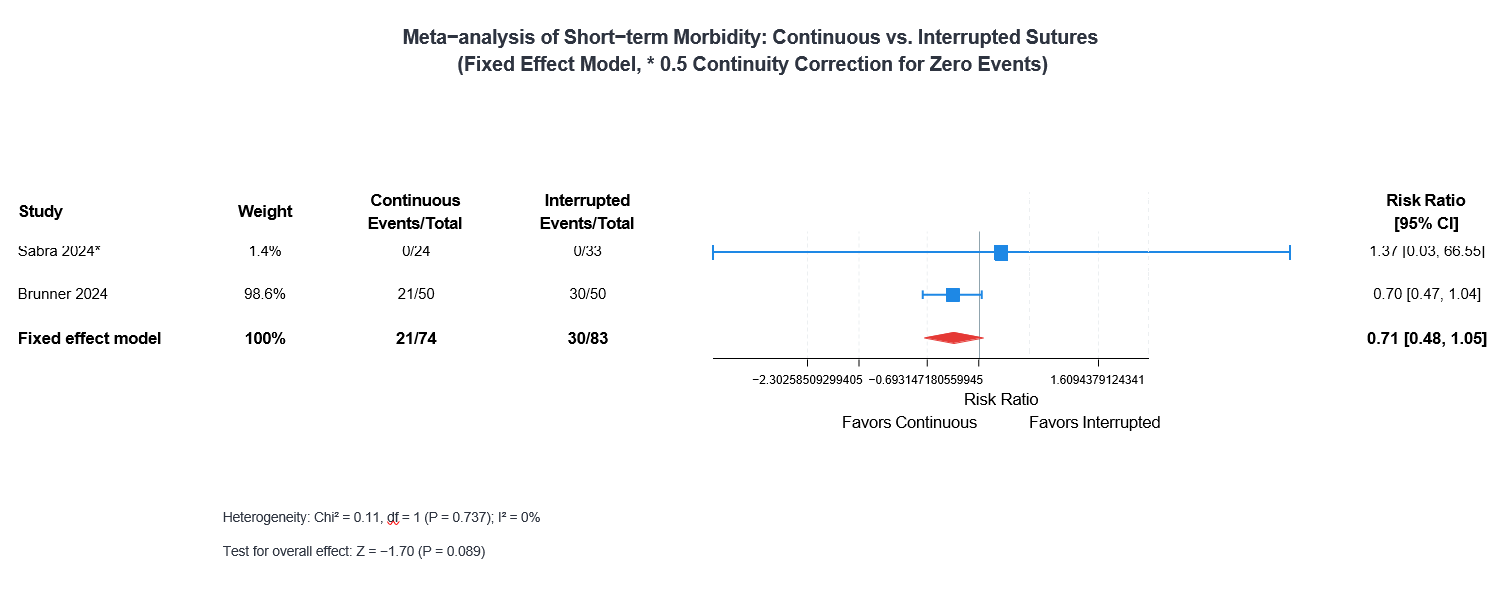
**

## Leave−One−Out Analysis of Short−term Morbidity (Fixed Effect Model, 0.5 Continuity Correction for Zero Events)

Short-term morbidity

### Analysis None (Overall)

Removed: Brunner 2018

Removed: Sabra 2024

Removed: Brunner 2024

### Risk Ratio [95% CI]

**0.78 [0.59, 1.03]**

0.71 [0.48, 1.05]

0.77 [0.59, 1.02]

0.97 [0.76, 1.24]

### P−value 0.078

0.089

0.068

0.815

**I² 0.2%**

0.0%

0.6%

0.0%

−2.30258509299405 −0.693147180559945 0 0.693147180559945 1.6094379124341

Risk Ratio (Mantel−Haenszel Fixed−Effect)

# Long-term Morbidity

# (Supplementary Fig.8)

## Meta−analysis of Overall Morbidity: Continuous vs. Interrupted Sutures (0.5 Continuity Correction for Zero Events Only)

**Study**


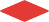


Saxane 2021

70.5%

34/88

200/468

0.90 [0.68, 1.20]

Natsume 2021

21.0%

23/116

14/56

0.79 [0.44, 1.42]

Seifert 2022

8.0%

3/41

7/39

0.41 [0.11, 1.47]

Sabra 2024

0.5%

0/24

0/33

1.37 [0.03, 66.55]

**Overall**

**100%**

**60/269**

**221/596**

**0.84 [0.66, 1.08]**

**Weight**

**Continuous Events/Total**

**Interrupted Events/Total**

**Risk Ratio [95% CI]**

Heterogeneity: Chi² = 1.56, df = 3 (P = 0.669); I² = 0% Test for overall effect: Z = −1.34 (P = 0.181)

−2.30258509299405 0 1.6094379124341

Risk Ratio (Mantel−Haenszel Fixed−Effect) Favors Continuous Favors Interrupted

## Leave−One−Out Analysis of Overall Morbidity (0.5 Continuity Correction for Zero Events Only)

### Analysis None (Overall)

Removed: Saxane 2021

Removed: Natsume 2021

Removed: Seifert 2022

Removed: Sabra 2024

### Risk Ratio [95% CI]

**0.84 [0.66, 1.08]**

0.70 [0.41, 1.18]

0.86 [0.65, 1.13]

0.88 [0.68, 1.14]

0.84 [0.65, 1.08]

### P−value 0.181

0.178

0.272

0.330

0.175

**I² 0.0%**

0.0%

0.0%

0.0%

0.0%

−2.30258509299405 −0.693147180559945 0 0.693147180559945 1.6094379124341

Risk Ratio (Mantel−Haenszel Fixed−Effect)

# Re-Exploration Rate

# (Supplementary Fig.9)

## Meta−analysis of Re−Exploration Rates: Continuous vs. Interrupted Sutures (Fixed Effect Model, * 0.5 Continuity Correction for Zero Events)

**Study**

Saxane 2021* Seifert 2022 Yadav 2024* Brunner 2024

**Fixed effect model**

**Weight**

18.5%

23.9%

11.0%

46.6%

**100%**

**Continuous Events/Total**

0/88

3/41

1/16

2/50

**6/195**

**Interrupted Events/Total**

2/468

1/39

0/18

2/50

**5/575**

**Risk Ratio [95% CI]**

1.06 [0.05, 21.87]

2.85 [0.31, 26.28]

3.36 [0.15, 77.02]

1.00 [0.15, 6.82]

**1.71 [0.53, 5.51]**

Heterogeneity: Chi² = 0.78, df = 3 (P = 0.855); I² = 0% Test for overall effect: Z = 0.90 (P = 0.366)

0.10 0.20 0.50 1.0 2.0 5.00 10.00

Risk Ratio

Favors Continuous Favors Interrupted

## Leave−One−Out Analysis of Re−Exploration Rates (Fixed Effect Model, 0.5 Continuity Correction for Zero Events)

### Analysis None (Overall)

Removed: Saxane 2021

Removed: Seifert 2022

Removed: Yadav 2024

Removed: Brunner 2024

### Risk Ratio [95% CI]

**1.71 [0.53, 5.51]**

1.86 [0.52, 6.65]

1.36 [0.34, 5.48]

1.51 [0.42, 5.39]

2.34 [0.51, 10.66]

### P−value 0.366

0.338

0.670

0.526

0.273

**I² 0.0%**

0.0%

0.0%

0.0%

0.0%

−2.30258509299405 −0.693147180559945 0 0.693147180559945 1.60943791243412.30258509299405

Risk Ratio (Mantel−Haenszel Fixed−Effect)
